# Genomic variations and association study of agronomic traits in flax

**DOI:** 10.1186/s12864-018-4899-z

**Published:** 2018-07-03

**Authors:** Dongwei Xie, Zhigang Dai, Zemao Yang, Qing Tang, Jian Sun, Xue Yang, Xixia Song, Ying Lu, Debao Zhao, Liguo Zhang, Jianguang Su

**Affiliations:** 1 Institute of Bast Fiber Crops, Chinese Academy of Agricultural Sciences, Changsha, China; 2 The Institute of Industrial Crops, Heilongjiang Academy of Agricultural Sciences, Harbin, China; 3 Agricultural College, Northeast Agricultural University, Harbin, China; 4 Sino-Russian Agricultural Scientific and Technological Cooperation Center, Heilongjiang Academy of Agricultural Sciences, Harbin, China

**Keywords:** Flax, SLAF-seq, Genetic diversity, Evolution, GWAS

## Abstract

**Background:**

Flax (*Linum usitatissimum*. L) is an ancient oilseed and natural fiber crop. It could be divided into three categories by use, namely oil flax, fiber flax and oil-fiber dual purpose (OF). Cultivated flax is widely used in the food and textile industry. It is of great significance to elucidate the genetic characteristics of flax collections for accelerating the process of breeding improvement in this dual purpose crop. With the development of next-generation sequencing, we can use new methods, such as SLAF-seq (specific-locus amplified fragment sequencing), to decode unknown genomes of species. In this study, a high-through sequencing of flax collections using SLAF-seq was conducted. The evolutionary tendency was defined and candidate genes associated with agronomic traits of flax species were identified by Genome-Wide Association Studying (GWAS).

**Results:**

A flax collection consisting of 224 varieties were sequenced by SLAF-seq. In total, 346,639 SLAF tags were developed from all accessions, with an average sequencing depth of 7.19 for each accession. A total of 584,987 SNPs (single nucleotide polymorphism) with an MAF > 0.05 were identified from these SLAFs. The population structure division and phylogenetic analysis indicated a strong divergence among three kinds of flax groups. The genome-wide variation uncovered that oil flax had the highest genetic diversity and was considered to be the ancestor of fiber flax and oil-fiber flax. Sixteen associated peak SNPs for six traits were obtained by GWAS of oil-related traits using EMMAX (efficient mixed-model association eXpedited). Candidate genes and their related pathway were evaluated. A new GWAS was developed for fiber properties using the GLM (General linear model) model and a number of loci were identified.

**Conclusions:**

To our knowledge, this is the first study on discovery multiple loci for important agronomic traits of flax species using GWAS strategy. These results will provide the highest possibility of incorporating both high fiber and good oil traits in a single variety.

**Electronic supplementary material:**

The online version of this article (10.1186/s12864-018-4899-z) contains supplementary material, which is available to authorized users.

## Background

Flax (*Linum usitatissimum*. L), as an ancient oilseed and natural fiber crop, has been used by humans for more than 10,000 years in ancient Egypt and Sumeria [1]. Flax is an annual crop species, whereas the wild forms can also be biennial or perennial. Cultivated flax having three types, one is grown for oil (linseed), the other for fiber (fiber flax) and the third for dual purpose. Fiber flax is unbranched, whereas linseed (oil flax) is much shorter and highly branched. It was well utilized for various purposes, such as the production of edible oil, textiles fiber, animal feed and other industrial products [2]. The oil flax contains two essential fatty acids, alpha-linolenic acid (ALA) and linoleic acid (LA). ALA is a precursor for the synthesis of the long-chain polyunsaturated fatty acids, eicosapentaenoic acid (EPA) and docosahexaenoic acid (DHA) which are required for the development of nervous system [3]. Flax fiber provides source materials for textile industry and was used for a long history of human being. Therefore, it is of great significance to elucidate the genetic diversity of flax collections for crop improvement to dual purpose crop.

The *L. usitatissimum* is a diploid with *n* = 15 chromosomes [4]. In past decades, genetic diversity assessments in flax were carried out using polymorphism markers such as RAPD (random-amplified polymorphic DNA), ISSR (inter simple sequence repeat), SSR (simple sequence repeats), IRAP (inter-retrotransposon amplified polymorphism) [5–8]. The 448 microsatellite markers were used to characterize the core collection of flax accessions [9]. A flax collection of 125 pale flax accessions and the Canadian flax core collection of 407 accessions were genotyped using 112 genome-wide simple sequence repeat markers [10]. It was also reported that the Canadian flax core collection of 390 accessions was genotyped with 464 simple sequence repeat markers and agronomic traits associated genomic region were mapped [11]. But most of these studies assessed with few markers and could not explain the genetic diversity of cultivated flax in detail. Up to recent studies, the genome of flax (*Linum usitatissimum*) was released by de novo assembling short shotgun sequence reads. It provides a reference genome sequence of flax species and gives us great convenience for marker development and gene discovery [12]. By high-throughput sequencing, we have the ability to construct a high-density genetic map using SLAF markers, developed by SLAF-seq [13]. The genetic map is valuable for clarifying evolution patterns of flax and discovering gene locus controlling agronomic traits.

The specific indications of selection are retained in crop genome during domestication, and this information can be interpreted by analyzing genome sequences. The initial use of flax has been debated all the time. It was proposed that flax was firstly used for fiber by archeological evidence [14]. However, a study of genetic diversity of the stearoyl-ACP desaturase II (sad2) locus from flax and pale flax (*L. angustifolium*) showed flax was first domesticated for oil, rather than fiber [15]. Thus, for a complete understanding of this domestication process, a population with diverse flax collections is needed to be genetically evaluated at the genome-wide level.

Target traits for flax improvement between oil types and fiber types are not same. Linseed oil content and number of the capsule are the most important traits for oil flax breeding [16], whereas fiber percentage, technical length and plant type (height, branch number, etc.) are the essential indexes for fiber flax breeding. Only a few genes had been clarified that had functions in controlling some of these traits. Two *FAD3* (fatty acyl desaturase 3) desaturase genes control the level of linolenic acid in flax seed. These two genes shared 95.4% identity and were proved to be the major genes controlling linolenic acid levels in flax [17]. SSR-based linkage map of flax was constructed and major QTLs (quantitative trait locus) that separately controlled linoleic acid, linolenic acid and palmitic acid were identified [18]. These studies are not enough to understand the genetic mechanism of fatty acid biosynthesis in flax. Except that, the genetic control of other agronomic traits was largely unknown. For the development of the dual purpose cultivars, we need to target the genomic regions controlling stem fiber and seed quality traits simultaneously. Therefore, it gives us a complete insight into genetic basis underlying complex traits in flax by GWAS for these agronomic traits in a large panel of varieties. GLM and mixed Linear model (MLM) are the most commonly used algorithmic models in GWAS. But the computational complexity of the two methods is enormous. To solve this problem, an efficient exact method which was referred to as EMMAX, could reduce the computational time for analyzing large GWAS data sets from years to hours [19].

In this study, we conducted a high-through sequencing of a flax collection with 224 varieties around the world and constructed a comprehensive map of flax genome variation. Based on these studies, the evolutionary tendency was defined by analyzing the patterns of diversity of flax collections. Candidate genes associated with important agronomic traits of flax species were identified by GWAS and their related pathways were analyzed further.

## Results

### Sequencing of flax accessions

**Fig. 1:**
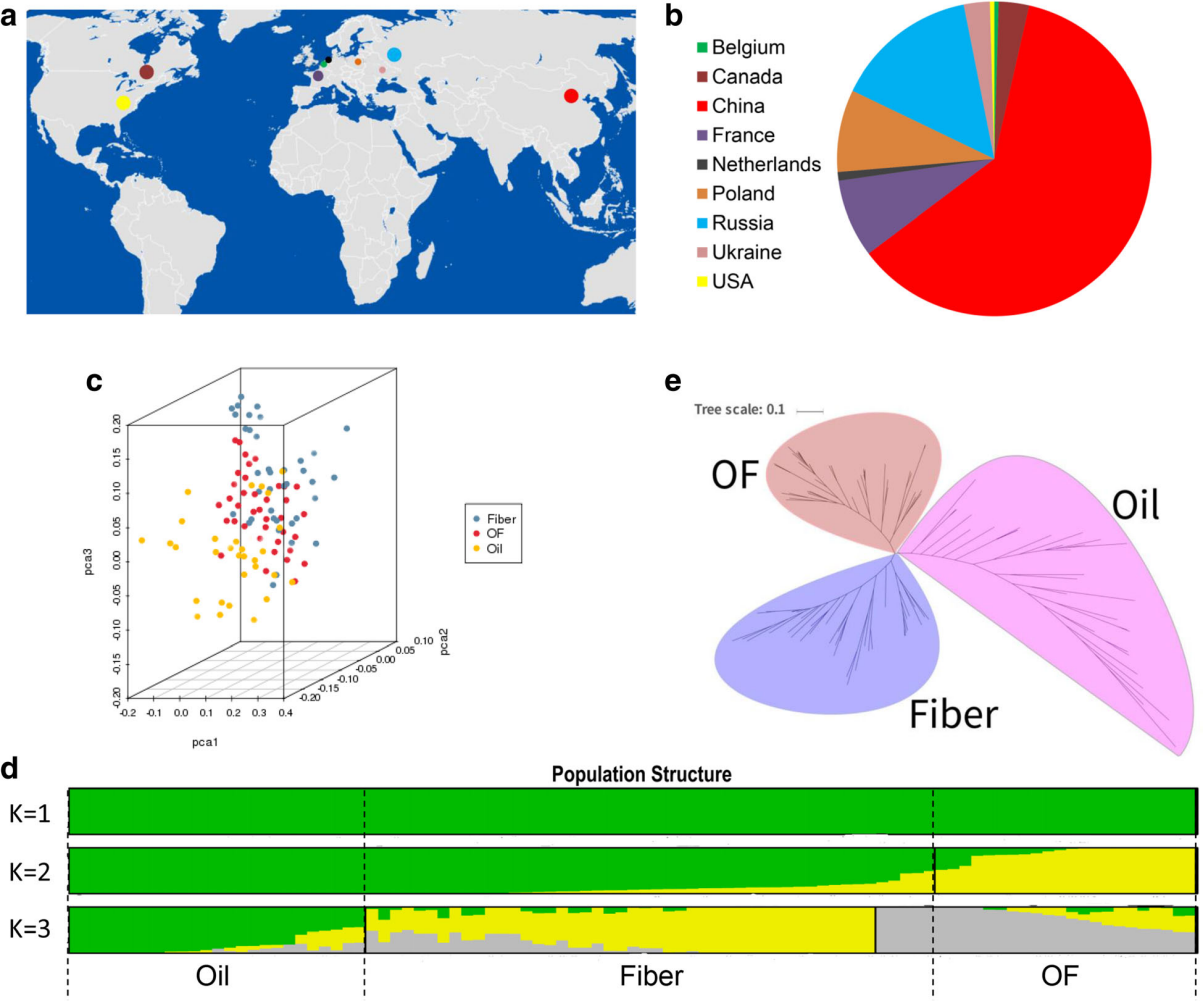
Sample distribution and divergence. **a** Worldwide distribution of flax accessions. **b** Proportion of materials in each geographical distribution. **c** Principal components analysis of flax samples. Each dot represents an accession. **d** Population structure of flax accessions. The accessions were divided into three groups: Oil flax group, Fiber flax group and Oil-fiber flax group (OF). **e** Phylogenetic tree of flax accessions

A total of 224 flax (*Linum usitatissimum* L.) accessions including oil seed flax type, fiber flax type and OF type were selected based on their phenotype and geographical distribution (Fig. 1a, b). By SLAF-seq method, 326.23 million reads were generated. The average high-quality base ratio (Q30) of all 224 accessions was 88.38%, and average GC (guanine-cytosine) content was 39.89% (Table 1). In total, 346,639 SLAF tags were developed from all accessions, with an average sequencing depth of 7.19 for each accession. The number of polymorphic SLAFs was 146,959. A total of 584,987 SNPs with an MAF > 0.05 were identified from these SLAFs (Table 2).

**Table 1:** Statistics of sequencing dat

Sample	Total reads number (Mb)	Q30^a^ Percentage (%)	GC Percentage (%)
224 accessions	326.23	88.38	39.89
Rice (*Nipponbare*)	0.75	87.86	42.79

^a^Q30 indicates a quality score of 30, indicating a 0.1% chance of error and thus 99.9% confidence

**Table 2:** Statistic results of SLAF tags and polymorphic markers

SLAF number	No. of polymorphic SLAF	Average depth	No. of polymorphic SNP
346,639	146,959	7.19	584,987

### Phylogenetic and population structure analyses

In order to ascertain the divergence of three kinds of flax species during evolution, we performed the principal component analysis. It showed that oil flax and fiber flax were clearly distinguished, but the oil-fiber group was mixed with fiber group (Fig. 1c). The results of population structure analyses showed that flax accessions were clearly divided into three groups as oil, fiber and OF groups at K = 3 (Fig. 1d). Further, phylogenetic analysis of 106 accessions showed that the oil, fiber and OF groups were clustered into three distinct branches (Fig. 1e). All these results indicated a strong divergence between different flax groups.

### Evolution patterns of flax species

**Fig. 2:**
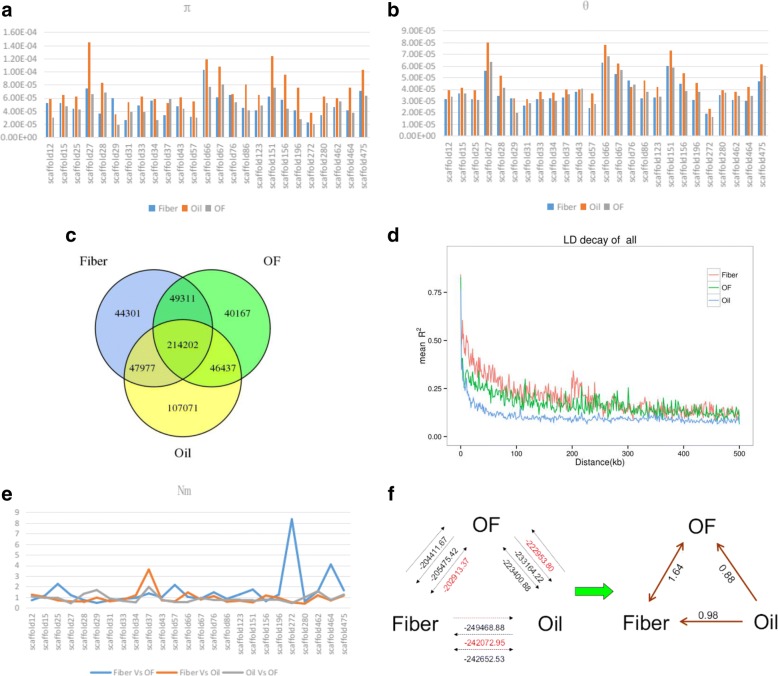
Evolutionary analysis of oil flax, fiber flax and oil-fiber flax. **a** and **b** The π and θ values of flax accessions. **c** Number of SNPs shared by oil flax, fiber flax and oil-fiber flax. **d** LD decay of three kinds of flax accessions. **e** The Nm (the number of migrants coming into the population) values of oil flax, fiber flax and oil-fiber flax. **f** Evolutionary route among three kinds of flax assumed by gene flow. All of the probable evolutionary patterns (dashed arrows) among three groups are summarized on the left figure, and the numbers on it represent the marginal likelihoods of each probable evolutionary pattern among three groups. High values of marginal likelihood suggest a high possibility of the corresponding pattern, and arrows point in the direction of the inferred gene flow. The proven evolutionary patterns are shown on the right. The numbers on the right of the figure are the Nm values reflecting the power of gene flow among the groups

As a domesticated crop, divergent selection for fiber flax and linseed flax resulted in a wide range of infraspecific variation. Genetic diversity of three kinds of flax was estimated by the nucleotide diversity (π) and Watterson estimator of θ within a population. Both π and θ values were higher in oil flax group compared with fiber flax group and dual-purpose group (Fig. 2a, b). Besides, the total and specific SNP numbers within groups were counted. It showed that oil flax group had the largest number of specific SNPs, while other two groups had a fair number of specific SNPs (Fig. 2c). These specific SNPs correlated with the maintenance of the distinguishable features of each group. The number of SNPs shared between any two groups was greater than that of their respective group-specific SNPs. This is sure because all three kinds of flax were domesticated from same ancient varieties. Moreover, Linkage disequilibrium (LD) analysis of these three groups revealed that the distance of LD decay in the oil flax group is shorter than those in the fiber flax and OF groups (Fig. 2d). All these results indicated that oil flax had a more diverse genomic background and considered to be the ancestor of fiber flax and oil-fiber flax.

To further elucidate the directions of domestication among the oil, fiber and OF groups, Nm values were used to measure gene flow rate among three groups. The results showed a relatively strong gene flow between Fiber group and OF group, but weak between Oil group and other two groups (Fig. 2e). The gene flow directions were analyzed using the Bayesian approach in MIGRATE to investigate the migration rates. As shown in Fig. 2f, oil flax is the ancient species and genetic elements flowed from oil flax to fiber flax and OF flax during domestication. It also indicated that gene flow between fiber flax and OF flax was bi-directional.

### Genome-wide association analysis of loci underlying oil traits by EMMAX model

There were many changes in the phenotype of flax during domestication, including plant type, process characters, yield traits, oil components and contents. Based on the 224 accessions, 13 agronomic traits were investigated for their phenotypic data in this study. Then, genome-wide association mapping was performed to analyze and identify the underlying genetic loci for these traits. Two distinct models, EMMAX and GLM were used for different traits association studies.

Above all, we used EMMAX model to compute the association signal of all 13 traits. The results showed that 16 associated peak SNPs were obtained for only six traits (T5-fruit number, T6-thousand grain weight, T9-the content of palmitic acid, T11- stearic acid content, T12- linoleic acid content, T13- linolenic acid content) (Table 3). Nonetheless, no peak SNPs in this model was detected for other 7 traits. Because LD patterns of three groups revealed that the distance of LD decay was about 10 kb (Additional file 1). So we selected 10 kb genomic regions around each peak SNP and identified candidate genes for each trait.

**Table 3:** Summary of peak SNPs of GWAS on 6 oil related traits by using EMMAX model

Trait	Chromosome (scaffold)	Physical position	*P* value
T5	scaffold346	438,191	0.00000041918
T6	scaffold43	1,111,162	0.0000011458
T6	scaffold51	598,586	0.00000051259
T6	scaffold51	598,611	0.00000047594
T6	scaffold51	699,833	0.00000027461
T6	scaffold261	925,068	0.000001073
T6	scaffold373	545,801	0.00000028845
T6	scaffold373	545,816	0.00000030249
T6	scaffold107	300,735	0.00000046771
T9	scaffold59	164,258	0.00000092704
T11	scaffold11	96,400	0.0000002192
T11	scaffold11	96,569	0.00000019606
T12	scaffold1253	27,622	0.00000039006
T13	scaffold416	80,582	0.00000004251
T13	scaffold302	224,377	0.00000020752
T13	scaffold302	224,395	0.00000020752

**Fig. 3:**
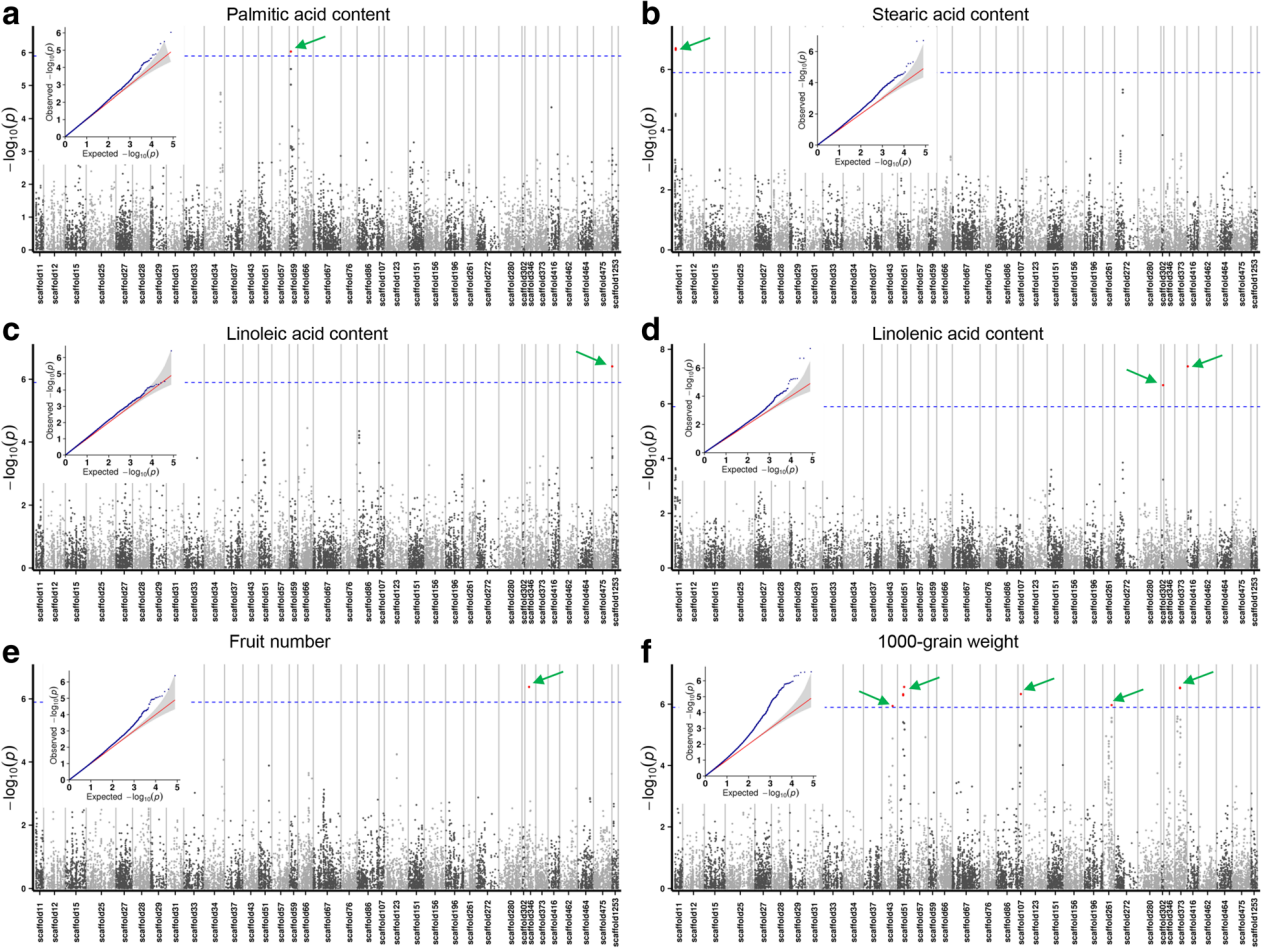
Genome-wide association study (GWAS) of agronomic traits in flax by using EMMAX model. **a**-**f** Manhattan plots with matching small QQ plots are shown. The peak SNPs are labeled by green arrows. The Bonferroni multiple test threshold is shown as a dash blue line (at *P* ≤ 0.01)

It is in commonly understood that the T9 and T11–13 were the bases for oil flax species. Therefore, we analyzed the candidate genes controlling these traits in depth. The peak SNP responsible for palmitic acid content was located at physical position 164,258 within the scaffold 59 (Fig. 3a, Table 3). In the candidate region near this peak SNP, one predicted gene- Lus10022606 encoded a phosphatidylinositol 4-phosphate 5-kinase (PIP5K) (Additional file 2). It was playing a role in the inositol phosphate metabolism pathway according to the KEGG (Kyoto Encyclopedia of Genes and Genomes) pathway maps (Additional file 3). Because hydrolysis of phosphatidylinositol can serve as precursors for the synthesis of palmitic acid, we considered lus10022606 as a candidate gene for palmitic acid biosynthesis.

Besides, the peak SNPs associated with stearic acid content located at position 96,400 and 96,569 in the scaffold 11 (Fig. 3b, Table 3). But the predicted genes around peak SNPs seemed to be unrelated to the fatty acid pathway (Additional file 2).

Linoleic acid and linolenic acid are two important unsaturated fatty acids in oil flax. The underlying genetic loci of these two compounds synthesis had also detected. One peak SNP at position 27,622 in the scaffold 1253 was a significant signal corresponding to the linoleic acid content (Fig. 3c, Table 3). There were three predicted genes in the designated candidate areas (Additional file 2). But we cannot directly determine the candidate gene based on the predicted information. According to genomic knowledge, we know that the biological functions of most of the genes are shared in eukaryotes, and even with prokaryotes [20]. Therefore, we developed gene ontology (GO) with three categories (biological process, molecular function and cellular component) to illuminate functions of the genes in the candidate region. Through GO annotation analysis, we found that the gene Lus10017450 is involved in the redox process of the organism (GO:0016491), which is a necessary step in the synthesis of linoleic acid. Therefore, we hypothesized that it may be a candidate gene for the control of linoleic acid synthesis.

In addition, three peak SNPs within two scaffolds were detected and considered to be responsible for the synthesis of linolenic acid (Fig. 3d, Table 3). However, it is very difficult to identify candidate genes based on gene prediction and GO annotations. None of the candidate genes near these peak SNPs seemed to be related to the linoleic acid or linolenic acid biosynthesis pathway (Additional file 2). Additional evidence was needed to support the identification of candidate genes.

### Exploring loci for fiber-related traits by using GLM model

Owing to the limit of EMMAX model in detecting loci responsible for plant height and fiber output which are important traits for flax fiber breeding, we further used the GLM model to perform computations.

**Fig. 4:**
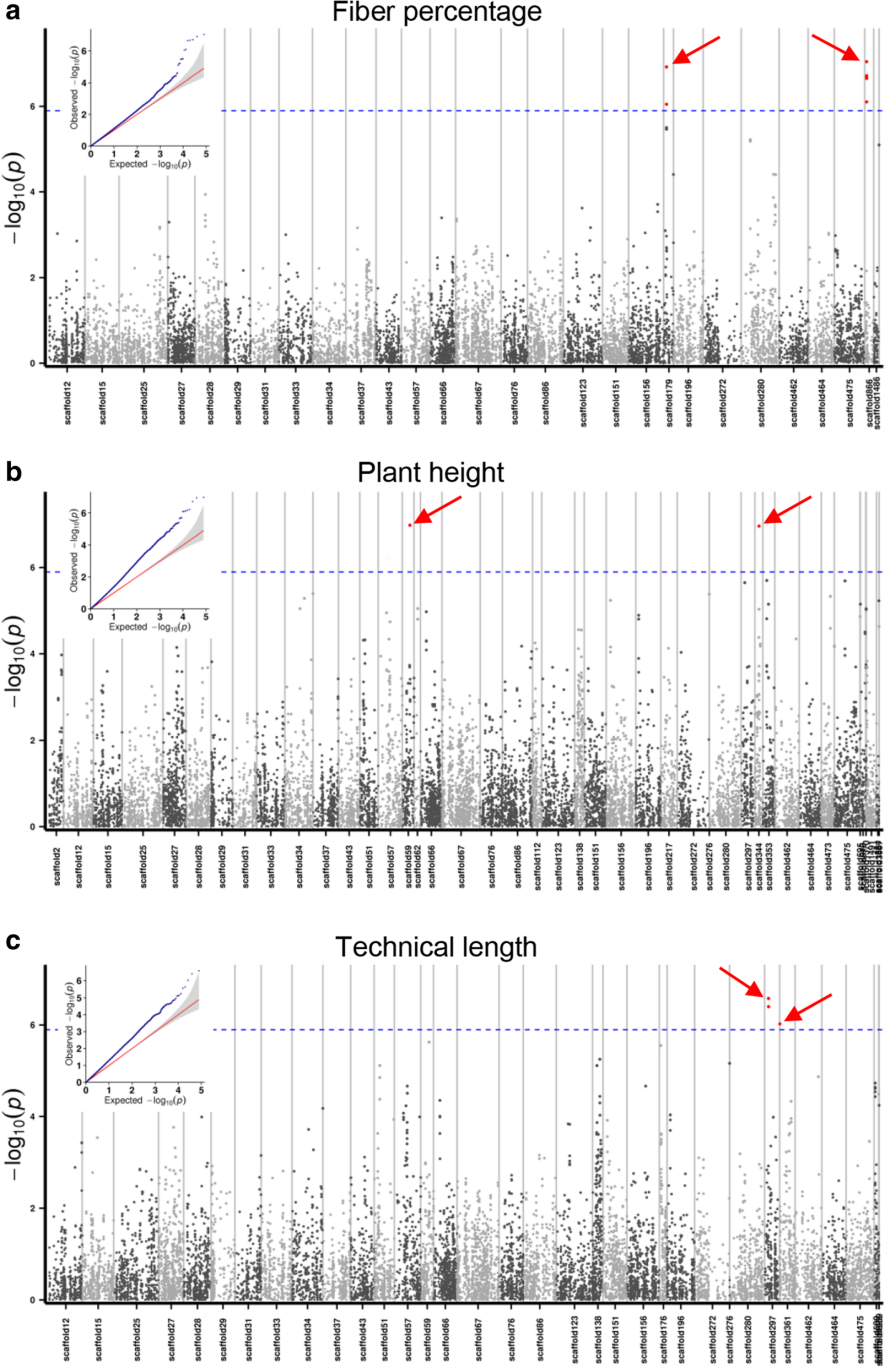
Genome-wide association study (GWAS) of agronomic traits in flax by using GLM model. **a**-**c** Manhattan plots with matching small QQ plots are shown. The peak SNPs are labeled by red arrows. The Bonferroni multiple test threshold is shown as a dash blue line (at P ≤ 0.01)

As a result, several loci controlling fiber percentage (T1), plant height (T2) and technical length (T3) were identified, respectively. Two distinct loci contributing to fiber percentage were found in scaffold 179 and scaffold 866 (Fig. 4a, Table 4). Peak SNP in scaffold 179 was located at physical position 179,593, and was in the UTR (untranslated region) of the predicted gene lus10016354. This candidate gene encodes a xanthoxin dehydrogenase which involved in the synthesis of ABA (abscisic acid) (Additional file 4). As reported before, ABA can reduce the synthesis of hemicellulose and cellulose [21], and *KOB1*, associated with ABA, can cause less crystalline cellulose [22, 23]. So we suggest that lus10016354 is a causal gene for fiber percentage in flax species.

**Table 4:** Summary of peak SNPs of GWAS on fiber traits by using GLM model

Trait	Chromosome (scaffold)	Physical position	*P* value
T1	scaffold179	179,593	0.00000012181
T1	scaffold866	116,645	0.000000091811
T2	scaffold344	309,662	0.00000011126
T2	scaffold59	572,553	0.0000001059
T3	scaffold297	275,113	0.0000003962
T3	scaffold297	275,131	0.00000026216
T3	scaffold361	14,957	0.000000944

By examining the inheritance of plant height, two significant loci were detected in scaffold 344 and scaffold 59 (Fig. 4b, Table 4). In the candidate region around the peak SNP at position 309,662, one gene Lus10016125 encoding ABC transporter (ATP-binding cassette transporters) was found (Additional file 4). As we known, ABC transporter had been largely identified and played a vital role in plant height development [24, 25]. Hence, we speculate this gene to be the candidate gene for flax height.

Technical length is positively related to the plant height to some extent, but the genetic loci responsible for these two traits are quite different. We detected two loci which located in scaffold 297 and scaffold 361 (Fig. 4c, Table 4). But it was difficult to determine the candidate genes under existing GWAS results.

In all, we have systematically studied the genetic mechanisms and identified a number of loci or candidate genes controlling each trait. These results will be helpful for further studies to discover the molecular mechanism of these traits.

### RNA-seq verification

To verify our GWAS results from expression level, we conducted transcriptome analysis of two oil content contrasting genotypes, high oil content accession Lu194 and low oil content accession Lu59 (Additional file 5), and found that the gene *Lus10021171* located nearby peak SNPs were highly expressed in Lu194 than that in Lu59 (Table 5). It encodes a protein phosphatase. But the stearic acid content in Lu194 is lower than that in Lu59. So this gene may play a negative role in stearic acid synthesis. Besides, candidate locus related to 1000- grain weight were also detected by using EMMAX model (Additional file 2). Among these QTLs, only the gene Lus10014560 which encodes a guanine nucleotide-binding protein was differentially expressed between Lu194 and Lu59 (Table 5). The higher expression of *Lus10014560* in Lu194 seems to be responsible for its higher 1000- grain weight.

**Table 5:** Candidate genes with differentially expressed patterns between Lu194 and Lu59

Phenotype	Scaffold	#ID	FDR	log2FC	regulated
T11	11	Lus10021171	0.00269	1.115	up
T6	107	Lus10014560	0.00009	2.105	up

## Discussion

### A feasible sequencing method to analyze the genetic diversity of flax species

Getting whole genome sequences is a crucial step in mining useful genetic information. Currently, it is difficult to obtain flax genome sequences based on chromosome-level positions and we have to use the less satisfactory reference genome sequence of flax (CDC Bethune), which has been assembled through whole-genome shotgun method and sequence assembly completion is relatively low with an estimated 81% genome coverage [12]. In this study, we carried out SLAF sequencing for 224 accessions and reads mapping using the genome sequence of CDC Bethune. As a result, low genome coverage of the reference reduced the called SNPs and thus limited GWAS mapping efficiency. Limited annotation information also sets up barriers on candidate gene screening. Furthermore, our RNA-seq experiment detected only two posible candidate genes (Table 5). However, it still helped us to a great extent to make genetic evolution analysis and gene mapping for flax species and we think a better reference will lead to a better mapping results and candidate gene set.

Within the genome-wide range, we found 584,987 useful SNPs. This indicates that there is an average SNP per 1.2 kb at the genome level. For a species without fine genome sequence, the marker density is already high enough to complete the GWAS and other associated studies. We selected materials mainly from China, Europe and North America where are the main geographical areas for flax growing, so these accessions were well represented. Judging from the results of SLAF sequencing, the main index, such as Q30 and sequencing depth, met the requirements for further studies [26, 27]. Practically, the SLAF sequencing strategy has been highly effective in SNP discovery for species from a rang of geographic location.

### Evaluation of origin and evolution of flax at genome level

It is very important to study the genetic diversity of crop species which can provide valuable data and information for breeding and germplasm innovation. In the present study, we clarified that oil flax was an ancient species. This is consistent with previous studies on the *sad2* (sized stearoyl-ACP desaturase II) gene [15]. But when we looked into the evolutionary relationship between fiber flax and oil-fiber flax, we found that the evolutionary pathway was uncertain and lacked the support of genetic data. As shown in Fig. 2f, we found a tendency for gene flow between oil group and other two groups, but in fact it was not significant between oil group and oil-fiber group. Furthermore, inferred gene flow between fiber group and oil-fiber group was bi-directional. So we were only able to determine that evolutionary process originated from oil group, but could not illustrate the exact evolutionary pathway among three groups.

As we know, nucleotide diversity of oil flax is higher than that in the other two groups (Fig. 2a, b). But the values of Tajima’s *D* and Fu & Li’s *D/F* were nearly same among three groups and were not significant (Additional file 6). These results indicated that domestication process from oil flax to fiber flax or oil-fiber flax was not under artificial selection or just underwent weak selection at a genome-wide level. In the other hand, the pale flax (*Linum bienne* Mill.) was considered to be the wild progenitor of cultivated flax (*Linum usitatissimum*) [28]. It is also suggested that the strong genetic differentiation occurred between cultivated and pale flax [10, 29]. A thorough analysis of the origin and evolution of flax requires simultaneous study of ancestral species, with the purpose of analyzing the evolutionary characteristics of cultivated flax in a larger context. This helps us to see whether or not oil flax and fiber flax originated from their ancestors independently.

Anyhow, in this study we conclude that oil flax is an ancient flax species and it has a higher genetic diversity. This provides basic knowledge for development and utilization of new oil flax resources in the breeding process.

### QTL analysis

As mentioned above, some QTLs controlling fruit number (T5) and 1000-grain weight (T6) have been detected, respectively. It has been reported that fruit number has the most direct influence on flax yield [16]. As a consequence, we found only one fruit number controlling QTL which locates in scaffold 346 with the peak SNP at position 438,191 (Fig. 3e, Table 3). Meanwhile, five distinct QTL responsible for 1000- grain weight were detected (Fig. 3f, Table 3). It indicates that 1000-grain weight is controlled by multiple loci. In addition, by analyzing a recombinant inbred line population of 243 individuals, only one QTL for 1000- grain weight was detected [30]. So it is a powerful method to explore QTLs by analyzing flax accessions using SNP markers.

Lignan is an important metabolic component in flaxseed, but the related QTL that controls its synthesis has not been detected in the two models. This may be due to differences in genetic background and environmental effects of the studied population. More accessions, higher molecular marker density and multi-plot trials for several years will always generate satisfied results in QTL resolution, QTL numbers and QTL true positives [31–33]. Though SLAF-seq is an efficient method for SNP discovery and GWAS [34, 35], a re-sequencing method can mine more SNPs and may make a difference for lignan QTL identification. Also, multiple location trial will benefit the QTL results of all the 13 traits.

### Models of GWAS on gene exploration in flax

Many model algorithms are available to address false positives and QTL detection rate. GLM (Q) is a traditional method for GWAS and it emphasis on dealing with false positives caused by population structure [36], while EMMAX model comprises kinship [37]. This study is the first to use high-density SNPs for GWAS in flax species. Two different models, EMMAX and GLM, were used and the similarities and differences between them were compared. In the GWAS results calculated by the EMMAX model, we only found QTLs which directly (T9, T11, T12, T13) or indirectly (T5 and T6) were related to oil traits. Further computation using GLM model revealed gene loci controlling fiber-related traits. However, the results calculated by this model were not satisfactory as shown in Quantile-Quantile plots. Lately, new model like GEMMA mvLMM [38] and FarmCPU [39] can handle joint multi-trait analysis and complex population structure GWAS, respectively. So based on the current sequencing data and phenotype data, more consistent QTLs may be detected. In any case, this paper provides important clues and references for genetic analysis of related traits in flax. In future work, population structure should be optimized for subsequent GWAS.

## Conclusions

In this study, we performed SLAF-seq for 224 flax accessions. A total 584,987 SNPs with an MAF > 0.05 were identified from the sequenced SLAFs. Genome-wide variation uncovered that the oil flax had the highest genetic diversity and was considered to be the ancestor of fiber flax and oil-fiber flax. Series of associated peak SNPs for agronomic traits were obtained by GWAS using EMMAX and GLM, respectively. To our knowledge, this is the first study on discovery of multiple loci for important agronomic traits of flax species using GWAS strategy. These results will not only provide the highest possibility of incorporating both high fiber and good oil traits in a single variety but will also open the new horizons for researchers and breeders.

## Methods

### Flax materials and phenotypes investigation

A total of 224 flax (*Linum usitatissimum* L.) accessions were used for this study. All the materials were selected from core collection with geographical distribution in China, Russia, Europe and North America, respectively. The type of each accession was listed (Additional file 7). Plants were grown in field condition in Harbin, China. Materials were planted in row with a line-spacing of 20 cm. Each accession was prepared for three replicates.

At maturation, ten vigorous guarded plants were selected from each replication for phenotyping. The 13 agronomic traits (T1, fiber percentage; T2, plant height; T3, technical length; T4, branch number; T5, fruit number; T6, 1000-grain weight, T7, lignan content; T8, total content of fatty acids; T9, palmitic acid content; T10, oleic acid; T11, stearic acid; T12, linoleic acid; T13, linolenic acid) of flax accessions were investigated. Fiber percentage was calculated as the percentage of long and short fiber weight obtained after processing to dry stem weight. The technical length was obtained by measuring the distance between cotyledon of the flax plant and the base of the first branch below inflorescence. Detailed data are available in Additional file 5.

For analysis of oil content, 20 g powdered flax seed samples were extracted under ultrasonic waves for 30 min at 60–90°Cwith eight times of petroleum ether. Each sample was extracted twice, and then the total fatty acids were recycled through pressure reduction. The 70 mg extracts of total fatty acids were diluted with 1 L 0.5 M methanolic KOH, followed by being heated in a water bath for 30 min at 60 °C. Then 1 mL methanol was added to the solution and heated in a water bath for 15 min at 60 °C. After cooling to room temperature, 1 mL normal octane was added and mixed thoroughly. By the addition of NaCl saturated solution, the extract was prepared for further detection. The sample extracts were analyzed using a gas chromatography (GC-14C, Shimadzu Company, Japan) equipped with a 2 m*3 mm column. The temperature of the column was 190 °C, while it was 250 °C in vaporizing room and detecting room. Each sample was detected and assayed in three replications and their mean value of peak areas was used to quantify the total oil content. Standard compounds of five kinds of fatty acids are purchased from the company of NU-CHEK.

### SLAF library construction and sequencing

Genomic DNA was isolated from the fresh leaves of the plants. The SLAF library was generated by using the method as described before with minor revision [13]. Genomic DNA of each sample was digested with HaeIII and Hpy166II. Then, the ATP and dual-index sequencing adapter were added at the 3′ and 5′ end of the digested DNA products, respectively [40]. PCR was performed and the products were purified using E.Z.N.A.H Cycle Pure Kit (Omega). The purified products were mixed and incubated with these two restricted enzymes again. After ligation of ATP, and Solexa adapter in the pair-end, the reaction products were purified using a Quick Spin column (Qiagen, Venlo, Netherlands), and segregated on a 2% agarose gel. Fragments with 350-450 bp were isolated using a Gel Extraction Kit (Tiangen). These SLAFs were subjected to PCR to add barcode 2. The PCR products were re-purified and then prepared for paired-end sequencing on an Illumina HiSeq 2500 sequencing platform (Illumina, San Diego, CA, USA).

### Data processing and SNP calling

All sequenced reads with clear index information were clustered according to sequence similarity.. The reads of all samples were mapped to the reference genome by BWA [41]. GATK was used for SNP calling [42]. The sequence accuracy rate was evaluated by using the rice variety-nipponbare as a control. Sequencing depths of each sample were calculated using the ‘Depth of Coverage’ module of GATK (the genome analysis toolkit) [42]. The SNPs in each SLAF were defined with our criteria (r^2^ > 0.8, MAF > 0.05).

### Phylogenetic and population structure analysis

The 80,667 SNPs of 106 accessions were obtained after filtration with the standard of an integrity > 0.5 and MAF > 0.05. All of these SNPs were genotyped by MEGA 6 (Molecular Evolutionary Genetics Analysis version 6) software and used for the construction of the phylogenetic tree by the neighbor-joining method [43].

Population structure of the flax species was calculated using Admixture software [44]. The analysis used 80,667 SNPs of 106 accessions to infer the genetic background of an accession that belongs to a cluster under a given number of populations (K). The number of genetic clusters was predefined as K = 1–5 for all accessions to explore the population structure.

### Genomic nucleotide diversity and LD decay analysis

Assessments of the population diversity were conducted by evaluating the levels of nucleotide variation (θ) and nucleotide diversity (π) of each group of flax species. The 80,667 SNPs from 106 accessions were selected to calculate the value of π and θ. The number of accessions in oil group, fiber group and OF group was 27, 34 and 34, respectively.

LD between pairs of SNPs was estimated by using squared allele frequency correlations (r^2^) in Tassel version 3.0 [45]. Only SNPs with an MAF more than 0.05 and less than 10% missing data were used.

### Estimation of gene flow

To estimate the average levels of gene flow, the Nm (number of migration) values were computed based on SNPs [46]. The directions of gene flow among three groups were estimated using Migrate [47]. Three models were used to evaluate their likelihoods, respectively. 1) A full model with two population sizes and two migration rates (from popA to popB and from popB to popA); 2) A model with two population sizes and one migration rate to popB and 3) a model with two population sizes and one migration rate to popA. The marginal likelihoods of all three models were compared to infer the direction of gene flow.

### Genome-wide association analyses of agronomic traits

Total SNPs from 224 accessions were used for GWAS. The efficient mixed model was performed using EMMAX software and GLM was performed using Tassel software. Population structure matrix generated from Admixture was used as Q matrix to performe GLM model. The *P* ≤ 1.268*10^− 5^ ((*P* = 0.01/n; n = total markers used, which is roughly a Bonferroni correction, corresponding to -log10 (P) = 5, red line) and P ≤ 1.268*10^− 6^ (*P* = 0.1/n; n = total markers used, which is roughly a Bonferroni correction, corresponding to -log10 (P) = 6, blue line) were defined as genome-wide control threshold and suggestive threshold. The genes within 10 Kb of these significant SNPs flanking region were reported as candidate genes.

### Transcriptome verification analysis

Two flax accessions, Lu194 (high oil content) and Lu59 (low oil content), were selected for transcriptome analysis. The developing seeds at 10 days after flowering were harvested, immediately frozen in liquid nitrogen, then stored at − 80 °C until extraction of RNA was performed. Three biological replicates per treatment (each sample containing 10 plants each) were processed in parallel. For each sample, total RNA was extracted using the Spectrum Plant Total RNA Kit (Sigma-Aldrch). Next, absorbances of total RNA isolates were read using a NanoDrop™ Lite spectrophotometer and RNA was evaluated for size, quantity and quality using an Agilent 2100 Bioanalyzer. The Illumina HiSeq 2500 platform was used to generate transcriptome sequences containing paired-end (PE) raw reads of about 100 bp in length. Sequencing-received raw reads were transformed by base calling into sequence data. Prior to mapping the readings from the reference database, all sequences were filtered and obtained clean tags. The clean reads mapped in reference database from multiple genes were filtered. The remaining clean tags were used as unambiguous clean tags. The number of unambiguous clean tags for each gene was calculated and normalized to TPM (transcripts per million clean tags). We used a rigorous algorithm method to identify differentially expressed genes between two samples. False discovery rate (FDR) was applied to determine the threshold of *P*-value in multiple tests and analysis. The DEGs were obtained through FDR ≤0.005 and |log2Ratio| ≥1.

## Additional files

Additional file 1: **Figure S1.** LD decay of 224 flax accessions. (PNG 40 kb)

Additional file 2: **Table S1.** Predicted genes in the candidate regions by GWAS using EMMAX model. (DOCX 21 kb)

Additional file 3: **Figure S2.** Putative metabolic pathway associated with palmitic acid content. (PNG 29 kb)

Additional file 4: **Table S2.** Predicted genes in the candidate regions by GWAS using GLM model. (DOCX 15 kb)

Additional file 5: **Table S4.** Phenotypic data of 224 flax accessions. (XLS 122 kb)

Additional file 6: **Figure S3.** Neutrality tests. (TIF 1802 kb)

Additional file 7: **Table S3.** The information of 224 flax accessions. (XLSX 16 kb)

## Abbreviation

ABA: Abscisic acid
ABC: Transporter
ALA: Alpha-linolenic acid
ATP: Adenosine triphosphate
ATP: Binding cassette transporters sad2: sized stearoyl-ACP desaturase
BLAT: The BLAST-like alignment tool
DHA: Docosahexaenoic acid
DNA ISSR: Inter simple sequence repeat
EMMAX: Efficient mixed-model association eXpedited
EPA: Eicosapentaenoic acid
FAD3: Fatty acyl desaturase 3
FDR: False discovery rate
GATK: The genome analysis toolkit
GC: Guanine-cytosine
GLM: Generalized linear model
GO: Gene ontology
GWAS: Genome-wide association studying
II T1: Fiber percentage
IRAP: Inter-retrotransposon amplified polymorphism
KEGG: Kyoto encyclopedia of genes and genomes
KOH: Potassium hydroxide
LA: Linoleic acid
LD: Linkage disequilibrium
MAF: Minor allele frequency
MEGA 6: Molecular evolutionary genetics analysis version 6
MLM: Mixed Linear model
NaCl: Sodium chloride
OF: Oil-fiber dual purpose
PCR: Polymerase chain reaction
PE: Paired-end
PIP5K: Phosphatidylinositol 4-phosphate 5-kinase
QTL: Quantitative trait locus
RAPD: Random-amplified polymorphic
SLAF-seq: Specific-locus amplified fragment sequencing
SNP: Single nucleotide polymorphism
SSR: Simple sequence repeats
T10: Oleic acid
T11: Stearic acid
T12: Linoleic acid
T13: Linolenic acid
T2: Plant height
T3: Technical length
T4: Branch number
T5: Fruit number
T6: 1000-grain weight
T7: Lignan content
T8: Total content of fatty acids
T9: Palmitic acid content
TPM: Transcripts per million clean tags
UTR: Untranslated region

## References

[1] Zohary D, Hopf M, Weiss E. Domestication of plants in the old world: the origin and spread of domesticated plants in Southwest Asia, Europe, and the Mediterranean Basin. New York: Oxford university press on demand; 2012.

[2] Jhala AJ, Hall LM (2010). Flax (*Linum usitatissimum* L.): current uses and future applications. Aust J Basic Appl Sci.

[3] Uauy R, Peirano P, Hoffman D, Mena P, Birch D, Birch E (1996). Role of essential fatty acids in the function of the developing nervous system. Lipids.

[4] Ray C (1944). Cytological studies on the flax Genus, Linum. Am J Bot.

[5] Fu YB, Diederichsen A, Richards KW, Peterson G (2002). Genetic diversity within a range of cultivars and landraces of flax (*Linum usitatissimum* L.) as revealed by RAPDs. Genet Resour Crop Evol.

[6] Hüseyin U, Fu YB, Orhan K, Gregoryw P, Axel D, Peter K (2010). Genetic diversity of cultivated flax (*Linum usitatissimum* L.) and its wild progenitor pale flax (*Linum bienne* mill.) as revealed by ISSR markers. Genet Resour Crop Evol.

[7] Smýkal P, Bačovákerteszová N, Kalendar R, Corander J, Schulman AH, Pavelek M (2011). Genetic diversity of cultivated flax (*Linum usitatissimum* L.) germplasm assessed by retrotransposon-based markers. Theor Appl Genet.

[8] Soto-Cerda BJ, Maureira-Butler I, Muñoz G, Rupayan A, Cloutier S (2012). SSR-based population structure, molecular diversity and linkage disequilibrium analysis of a collection of flax (*Linum usitatissimum* L.) varying for mucilage seed-coat content. Mol Breed.

[9] Soto-Cerda BJ, Diederichsen A, Ragupathy R, Cloutier S (2013). Genetic characterization of a core collection of flax (*Linum usitatissimum* L.) suitable for association mapping studies and evidence of divergent selection between fiber and linseed types. BMC Plant Biol.

[10] Soto-Cerda BJ, Diederichsen A, Duguid S, Booker H, Rowland G, Cloutier S (2014). The potential of pale flax as a source of useful genetic variation for cultivated flax revealed through molecular diversity and association analyses. Mol Breed.

[11] Soto-Cerda BJ, Duguid S, Booker H, Rowland G, Diederichsen A, Cloutier S (2014). Genomic regions underlying agronomic traits in linseed (*Linum usitatissimum* L.) as revealed by association mapping. J Integr Plant Biol.

[12] Wang Z, Hobson N, Galindo L, Zhu S, Shi D, Mcdill J, Yang L, Hawkins S, Neutelings G, Datla R (2012). The genome of flax (*Linum usitatissimum*) assembled de novo from short shotgun sequence reads. Plant J.

[13] Sun X, Liu D, Zhang X, Li W, Liu H, Hong W, Jiang C, Guan N, Ma C, Zeng H (2013). SLAF-seq: an efficient method of large-scale *de novo* SNP discovery and genotyping using high-throughput sequencing. PLoS One.

[14] Zeist WV, Bakker-Heeres JAH (1975). Evidence for linseed cultivation before 6000 bc. J Archaeol Sci.

[15] Allaby RG, Peterson GW, Merriwether DA, Fu YB (2005). Evidence of the domestication history of flax (*Linum usitatissimum* L.) from genetic diversity of the sad2 locus. Theor Appl Genet.

[16] Copur O, Gur MA, Karakus M, Demirel U (2006). Determination of Correlation and Path Analysis among Yield Components and Seed Yield in Oil Flax Varieties (*Linum usitatissimum* L.). J Biol Sci.

[17] Vrinten P, Hu Z, Munchinsky MA, Rowland G, Qiu X (2005). Two FAD3 desaturase genes control the level of linolenic acid in flax seed. Plant Physiol.

[18] Cloutier S, Ragupathy R, Niu Z, Duguid S (2011). SSR-based linkage map of flax (*Linum usitatissimum* L.) and mapping of QTLs underlying fatty acid composition traits. Mol Breed.

[19] Kang HM, Sul JH, Zaitlen NA, Kong SY, Freimer NB, Sabatti C, Eskin E, Service SK (2010). Variance component model to account for sample structure in genome-wide association studies. Nat Genet.

[20] Ashburner M, Ball CA, Blake JA, Botstein D, Butler H, Cherry JM, Davis AP, Dolinski K, Dwight SS, Eppig JT (2000). Gene ontology: tool for the unification of biology. Nat Genet.

[21] Wakabayashi K, Sakurai N, Kuraishi S (1989). Role of the outer tissue in abscisic acid-mediated growth suppression of etiolated squash hypocotyl segments. Physiol Plant.

[22] Lertpiriyapong K, Sung Z (2003). The elongation defective1 mutant of Arabidopsis is impaired in the gene encoding a serine-rich secreted protein. Plant Mol Biol.

[23] Paredez AR, Persson S, Ehrhardt DW, Somerville CR (2008). Genetic evidence that cellulose synthase activity influences microtubule cortical array organization. Plant Physiol.

[24] Kushnir S, Babiychuk E, Storozhenko S, Davey MW, Papenbrock J, De RR, Engler G, Stephan UW, Lange H, Kispal G (2001). A mutation of the mitochondrial ABC transporter Sta1 leads to dwarfism and chlorosis in the Arabidopsis mutant starik. Plant Cell.

[25] Ye L, Liu L, Xing A, Kang D (2013). Characterization of a dwarf mutant allele of Arabidopsis MDR-like ABC transporter AtPGP1 gene. Biochem Biophys Res Commun.

[26] Han Y, Zhao X, Liu D, Li Y, Lightfoot DA, Yang Z, Zhao L, Zhou G, Wang Z, Huang L (2016). Domestication footprints anchor genomic regions of agronomic importance in soybeans. New Phytol.

[27] Huang X, Kurata N, Wei X, Wang ZX, Wang A, Zhao Q, Zhao Y, Liu K, Lu H, Li W (2012). A map of rice genome variation reveals the origin of cultivated rice. Nature.

[28] Diederichsen A, Hammer K (1995). Variation of cultivated flax (*Linum usitatissimum* L. subsp. *usitatissimum*) and its wild progenitor pale flax (subsp. *angustifolium* (Huds.) Thell.). Genet Resour Crop Evol.

[29] Uysal H, Kurt O, Fu YB, Diederichsen A, Kusters P (2012). Variation in phenotypic characters of pale flax (*Linum bienne* mill.) from Turkey. Genet Resour Crop Evol.

[30] Kumar S, You FM, Duguid S, Booker H, Rowland G, Cloutier S (2015). QTL for fatty acid composition and yield in linseed (*Linum usitatissimum* L.). Theor Appl Genet.

[31] Wang XL, Wang HW, Liu SX, Ferjani A, Li JS, Yan JB, Yang XH, Qin F (2016). Genetic variation in *ZmVPP1* contributes to drought tolerance in maize seedlings. Nat Genet.

[32] Fang L, Wang Q, Hu Y, Jia YH, Chen JD, Liu BL, Zhang ZY, G XY, Chen SQ, Zhou BL, Mei GF, Sun JL, Pan ZE, He SP, Xiao SH, Shi WJ, Gong WF, Liu JG, Ma J, Cai CP, Zhu XF, Guo WZ, Du XM, Zhang TZ (2017). Genomic analyses in cotton identify signatures of selection and loci associated with fiber quality and yield traits. Nat Genet.

[33] Varshney RK, Saxena RK, Upadhyaya HD, Khan AW, Yu Y, Kim C, Rathore A, Kim D, Kim J, An S, Kumar V, Anuradha G, Yamini KN, Zhang W, Muniswamy S, Kim J-S, Penmetsa RV, Wettberg EV, Datta SK (2017). Whole-genome resequencing of 292 pigeonpea accessions identifies genomic regions associated with domestication and agronomic traits. Nat Genet.

[34] Zhou QH, Han DP, Mason AS, Zhou C, Zheng W, Li YZ, Wu CJ, Fu DH, Huang YJ (2017). Earliness traits in rapeseed (*Brassica napus*): SNP loci and candidate genes identified by genome-wide association analysis. DNA Res.

[35] Li TG, Ma XF, Li NY, Zhou L, Liu Z, Han HY, Gui YJ, Bao YM, Chen JY, Dai XF (2017). Genome-wide association study discovered candidate genes of Verticillium wilt resistance in upland cotton (*Gossypium hirsutum* L.). Plant Biotechnol J.

[36] Wang M, Yan JB, Zhao JR, Song W, Zhang XB, Xiao YN, Zheng YL (2012). Genome-wide association study (GWAS) of resistance to head smut in maize. Plant Sci.

[37] Kang HM, Zaitlen NA, Wade CM, Kirby A, Heckerman D, Daly MJ, Eskin E. Efficient control of population structure in model organism association mapping.[J]. Genetics, 2008;178(3):1709. 10.1534/genetics.107.080101 PMC2278096 18385116

[38] Zhou X, Matthew S (2014). Efficient multivariate linear mixed model algorithms for genome-wide association studies. Nat Methods.

[39] Liu XL, Huang M, Fan B, Buckler ES, Zhang ZW (2016). Iterative usage of fixed and random effect models for powerful and efficient genome-wide association studies. PLoS Genet.

[40] Kozich JJ, Westcott SL, Baxter NT, Highlander SK, Schloss PD (2013). Development of a dual-index sequencing strategy and curation pipeline for analyzing amplicon sequence data on the MiSeq Illumina sequencing platform. Appl Environ Microbiol.

[41] Li H, Durbin R (2009). Fast and accurate short read alignment with Burrows-Wheeler transform. Bioinformatics.

[42] McKenna A, Hanna M, Banks E, Sivachenko A, Cibulskis K, Kernytsky A, Garimella K, Altshuler D, Gabriel S, Daly M, DePristo MA (2010). The genome analysis toolkit: a MapReduce framework for analyzing next-generation DNA sequencing data. Genome Res.

[43] Tamura K, Stecher G, Peterson D, Filipski A, Kumar S (2013). MEGA6: molecular evolutionary genetics analysis version 6.0. Mol Biol Evol.

[44] Alexander DH, Novembre J, Lange K (2009). Fast model-based estimation of ancestry in unrelated individuals. Genome Res.

[45] Bradbury PJ, Zhang Z, Kroon DE, Casstevens TM, Ramdoss Y, Buckler ES (2007). TASSEL: software for association mapping of complex traits in diverse samples. Bioinformatics.

[46] Hudson RR, Slatkin M, Maddison WP (1992). Estimation of levels of gene flow from DNA sequence data. Genetics.

[47] Beerli P, Palczewski M (2010). Unified framework to evaluate panmixia and migration direction among multiple sampling locations. Genetics.

