# Supplementary figures and images for: Genomic variations and association study of agronomic traits in flax
Source: BMC Genomics. 2018 Jul 3;19:512. doi: 10.1186/s12864-018-4899-z (PMC6029072; doi:10.1186/s12864-018-4899-z)

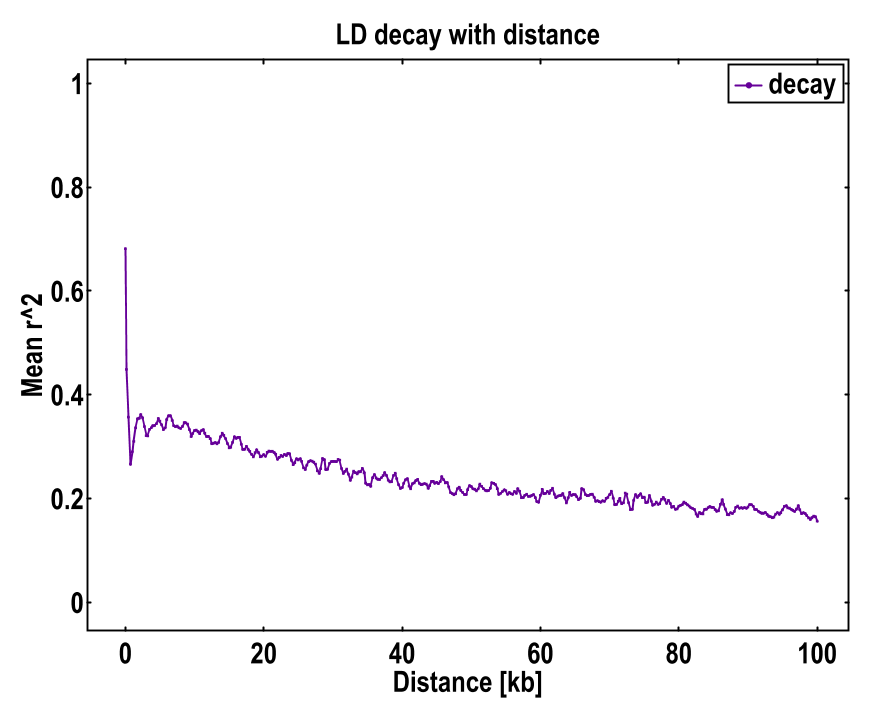

Supplement: Supplementary file 1 — Figure S1. LD decay of 224 flax accessions. (PNG 40 kb) [file 12864_2018_4899_MOESM1_ESM.png]

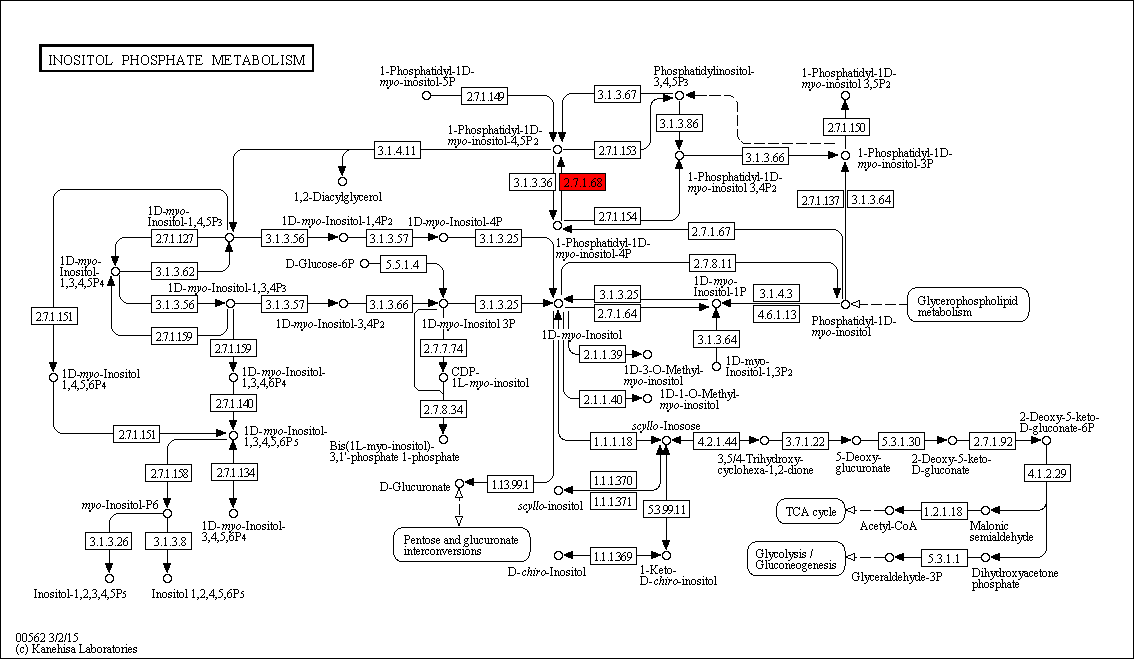

Supplement: Supplementary file 3 — Figure S2. Putative metabolic pathway associated with palmitic acid content. (PNG 29 kb) [file 12864_2018_4899_MOESM3_ESM.png]

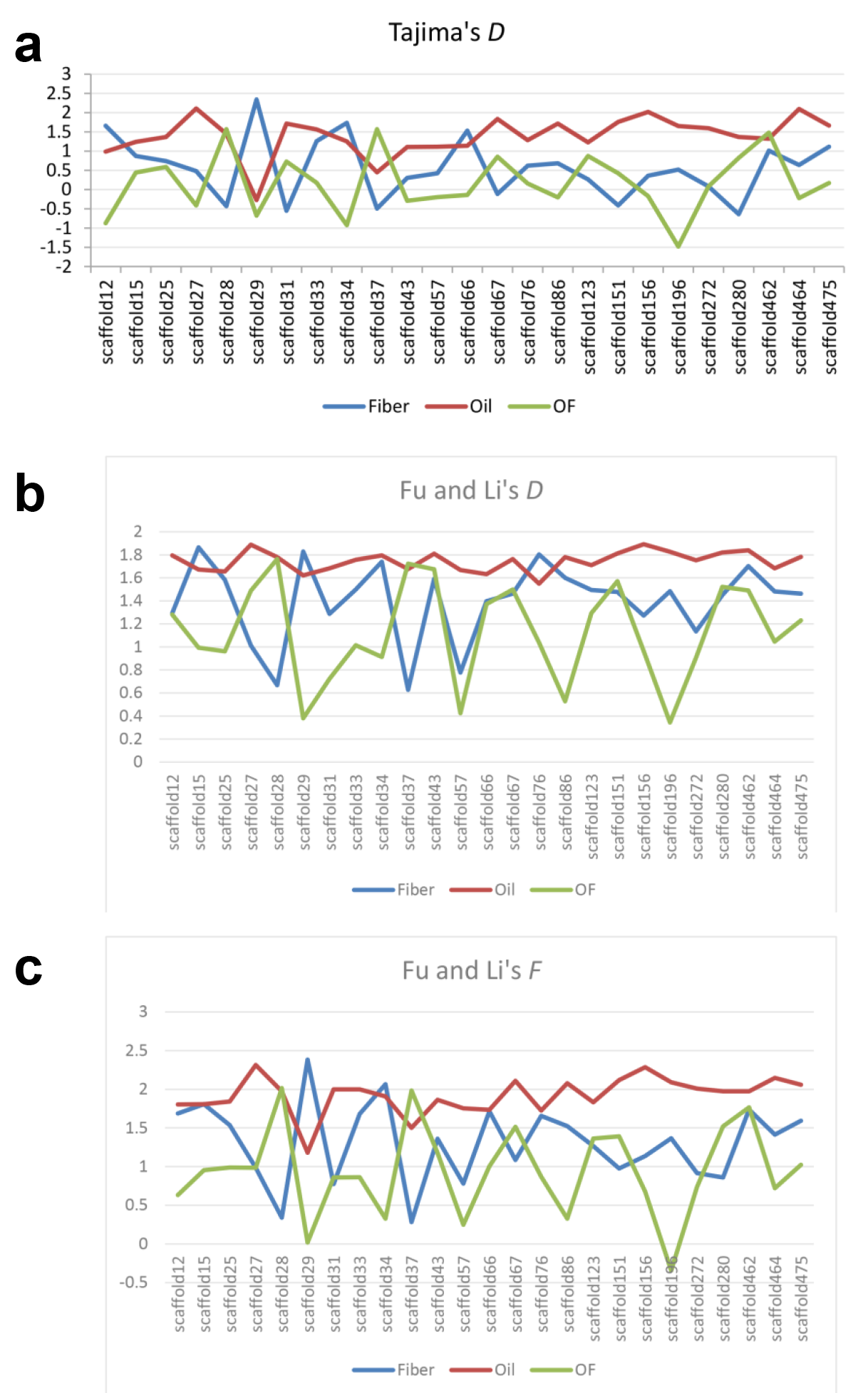

Supplement: Supplementary file 6 — Figure S3. Neutrality tests. (TIF 1802 kb) [file 12864_2018_4899_MOESM6_ESM.tif]
