# Supplementary material for: Genomic variations and association study of agronomic traits in flax
Source: BMC Genomics. 2018 Jul 3;19:512. doi: 10.1186/s12864-018-4899-z (PMC6029072; doi:10.1186/s12864-018-4899-z)
Supplement: Supplementary file 2 — Table S1. Predicted genes in the candidate regions by GWAS using EMMAX model. (DOCX 21 kb) [file 12864_2018_4899_MOESM2_ESM.docx]

| **Trait** | **Scaffold** | **Gene ID** | **Physical position** | **Predicted protein** |
| --- | --- | --- | --- | --- |
| T9 | scaffold59 | Lus10022606 | 152253-155508 | Phosphatidylinositol 4-phosphate 5-kinase |
|  |  | Lus10022607 | 155736-158074 | Uncharacterized protein |
|  |  | Lus10022608 | 159008-160048 | Ras-related protein |
|  |  | Lus10022609 | 160493-161783 | E3 ubiquitin-protein ligase |
|  |  | Lus10022610 | 161934-162602 | Zinc finger protein |
|  |  | Lus10022611 | 169658-170524 | Aquaporin TIP1-1 |
|  |  | Lus10022612 | 173529-175517 | Serine/threonine protein phosphatase |
| T11 | Scaffold11 | Lus10021170 | 95898-96954 | 60S ribosomal protein |
|  |  | Lus10021171 | 97962-99226 | Protein phosphatase |
|  |  | Lus10021172 | 99602-104865 | Uncharacterized protein |
| T12 | Scaffold  1253 | Lus10017450 | 18493-20324 | Uncharacterized protein |
|  |  | Lus10017451 | 22659-25147 | SEC12-like protein |
|  |  | Lus10017452 | 26799-33352 | Glycosyl transferases |
| T13 | Scaffold  302 | Lus10007372 | 211098-214756 | Sucrose synthase |
|  |  | Lus10007373 | 215517-216175 | Uncharacterized protein |
|  |  | Lus10007374 | 218224-218529 | Glucuronoxylan 4-O-methyltransferase |
|  |  | Lus10007375 | 219134-220253 | Uncharacterized protein |
|  |  | Lus10007376 | 221640-223464 | 40S ribosomal protein |
|  |  | Lus10007377 | 224278-227969 | NAC domain-containing protein |
|  | Scaffold  416 | Lus10029802 | 70679-73865 | serine/threonine-protein kinase |
|  |  | Lus10029803 | 76456-79227 | Uncharacterized protein |
|  |  | Lus10029804 | 80103-82835 | Polyamine oxidase |
|  |  | Lus10029805 | 86294-89595 | bHLH protein |
| T5 | Scaffold  346 | Lus10028621 | 427692-430524 | Uncharacterized protein |
|  |  | Lus10028622 | 432302-435774 | Vacuolar sorting-associated protein |
|  |  | Lus10028623 | 439983-442712 | Glycosyl transferase |
|  |  | Lus10028624 | 443490-445090 | Uncharacterized protein |
| T6 | scaffold43 | Lus10035211 | 1102028-1104095 | Mitochondrial import receptor subunit |
|  |  | Lus10035212 | 1107272-1111789 | Uncharacterized protein |
|  |  | Lus10035213 | 1112553-1115084 | Sulfite oxidase |
|  |  | Lus10035214 | 1116196-1117439 | SWI/SNF complex SNF12 homolog |
|  |  | Lus10035215 | 1117550-1118026 | SWI/SNF complex SNF12 homolog |
|  | scaffold51 | Lus10032962 | 605390-606559 | 60S ribosomal protein |
|  |  | Lus10032966 | 650858-690359 | Uncharacterized protein |
|  |  | Lus10032968 | 696537-698019 | Uncharacterized protein |
|  | Scaffold  261 | Lus10031062 | 913820-916718 | Ubiquitin carboxyl-terminal hydrolase |
|  |  | Lus10031063 | 918007-921173 | ABC transporter |
|  |  | Lus10031064 | 927563-931870 | 2OG-Fe(II) oxygenase |
|  |  | Lus10031065 | 933058-940429 | Uncharacterized protein |

**Table S1.** **Predicted genes in the candidate regions by GWAS using EMMAX model.**

**(Continued in next page)**

| **Trait** | **Scaffold** |  | |  | **Predicted protein** |
| --- | --- | --- | --- | --- | --- |
| **(Continued)** | | | |  |  |
| **Trait** | **Scaffold** | | **Gene ID** | **Physical position** | **Predicted protein** |
| T6 | Scaffold  373 | | Lus10030783 | 535850-536200 | Uncharacterized protein |
|  |  |  | Lus10030784 | 540144-542404 | Uncharacterized protein |
|  |  |  | Lus10030785 | 547425-549895 | serine/threonine-protein kinase |
|  |  |  | Lus10030786 | 550413-551358 | Ubiquitin-conjugating enzyme E2 |
|  |  |  | Lus10030787 | 552439-554307 | Pentatricopeptide repeat-containing |
|  |  |  | Lus10030788 | 554431-555929 | SNF1-related protein kinase |
|  | Scaffold  107 | | Lus10014558 | 295070-297364 | Dynein assembly factor |
|  |  |  | Lus10014559 | 297863-298405 | Oleosin |
|  |  |  | Lus10014560 | 299022-302339 | Guanine nucleotide-binding protein |
|  |  |  | Lus10014561 | 303613-306632 | GPI transamidase component |
|  |  |  | Lus10014562 | 308244-310764 | Heterogeneous ribonucleoprotein |
