# Supplementary material for: Genomic variations and association study of agronomic traits in flax
Source: BMC Genomics. 2018 Jul 3;19:512. doi: 10.1186/s12864-018-4899-z (PMC6029072; doi:10.1186/s12864-018-4899-z)
Supplement: Supplementary file 4 — Table S2. Predicted genes in the candidate regions by GWAS using GLM model. (DOCX 15 kb) [file 12864_2018_4899_MOESM4_ESM.docx]

**Table S2.** Predicted genes in the candidate regions by GWAS using GLM model.

| **Trait** | **Scaffold** | **Gene ID** | **Physical position** | **Predicted protein** |
| --- | --- | --- | --- | --- |
| T1 | scaffold179 | Lus10016353 | 172392-174854 | Vinorine synthase |
|  |  | Lus10016354 | 176312-179429 | Xanthoxin dehydrogenase |
|  |  | Lus10016355 | 180626-182602 | Uncharacterized protein |
|  |  | Lus10016356 | 183870-187487 | Inactive purple acid phosphatase |
|  |  | Lus10016357 | 188531-188972 | Protease inhibitor |
|  | scaffold866 | Lus10015118 | 110798-114443 | Glutamate receptor |
|  |  | Lus10015119 | 117833-119260 | Ras-related protein |
|  |  | Lus10015120 | 120383-120958 | Uncharacterized protein |
|  |  | Lus10015121 | 124753-128526 | Putative syntaxin |
| T2 | scaffold344 | Lus10016125 | 296918-300723 | ABC transporter |
|  |  | Lus10016126 | 301111-302651 | UDP-glycosyltransferase |
|  |  | Lus10016127 | 303894-305104 | UDP-glycosyltransferase |
|  | scaffold59 | Lus10022700 | 561437-564355 | Rho GTPase-activating protein |
|  |  | Lus10022701 | 567622-569256 | Uncharacterized protein |
|  |  | Lus10022702 | 569991-571616 | Uncharacterized protein |
|  |  | Lus10022703 | 573068-573775 | Uncharacterized protein |
|  |  | Lus10022704 | 574118-574476 | Uncharacterized protein |
|  |  | Lus10022705 | 580365-580789 | Replication protein |
|  |  | Lus10022706 | 581233-582780 | F-box/kelch-repeat protein |
| T3 | scaffold297 | Lus10027043 | 268309-269366 | Blue copper protein |
|  |  | Lus10027044 | 272010-273279 | Uncharacterized protein |
|  | scaffold361 | Lus10026704 | 15704- 17581 | Got1-like family protein |
|  |  | Lus10026705 | 18587- 19603 | Uncharacterized protein |
|  |  | Lus10026706 | 20494- 22964 | Tyrosine-protein kinase |
